# Genetic Diversity Based on Human Y Chromosome Analysis: A Bibliometric Review Between 2014 and 2023

**DOI:** 10.7759/cureus.58542

**Published:** 2024-04-18

**Authors:** Ramona Hodișan, Dana C Zaha, Claudia M Jurca, Codruta D Petchesi, Marius Bembea

**Affiliations:** 1 Doctoral School of Biomedical Sciences, University of Oradea, Oradea, ROU; 2 Department of Preclinical Disciplines, University of Oradea, Faculty of Medicine and Pharmacy, Oradea, ROU

**Keywords:** genetic diversity, human, population genetics, bibliometric analysis, y-chromosome

## Abstract

The Y chromosome has gained significant importance in the examination of genetic studies of populations because of its non-recombinant character and its form of uniparental inheritance. This work seeks to offer a comprehensive review of the specialty literature in the field of population genetics, focusing specifically on the analysis of the human Y chromosome using a bibliometric approach and knowledge mapping. This involves establishing worldwide structural networks by identifying the primary research themes, authors, and papers that have had a significant impact on the academic community. The objective is to examine global publications by analyzing citations at both the document and country level. This will involve conducting co-citation analysis for references with a high number of citations, examining bibliographic coupling through journal analysis, analyzing the co-occurrence of keywords, and investigating collaboration between authors from a country perspective. The research papers have been extracted from the Web of Science database. The bibliometric analysis was performed using the Bibliometrix and VOSviewer software tools. The purpose of this article is to serve as a starting point for future research dedicated to the analysis of the diversity of human Y chromosome haplotypes. The objectives of the study were to identify and present the most cited publications and references with the highest number of citations, and to highlight current publications at the national level.

## Introduction and background

The analysis of the Y chromosome has become an essential element in the interpretation of genetic studies of populations due to its non-recombining nature and the phenomenon of uniparental inheritance [1]. The Y chromosome has a segment that does not undergo recombination with the X chromosome during meiosis, and approximately 95% of the regions that cannot be exchanged or recombined are known as the non-recombining region of the Y chromosome (NRY) [2]. The main methods that analyze genetic diversity are the analysis of short tandem repeat polymorphism on the Y chromosome (Y-STR) and single nucleotide polymorphisms on the Y chromosome (Y-SNP) [3]. Y-SNPs are characterized by slow mutations, with the potential to be used in forensics, ancestral inference studies, and the investigation of human evolution [4]. The combined analysis of Y-STRs and Y-SNPs is highly beneficial in forensic applications and for constructing population genetic profiles. This is due to the fact that the mutation rate of Y-STRs ranges from 3.78 × 10^−4^ to 7.44 × 10^−2^ mutations per generation, while the Y-SNP mutation ratio is 1 × 10^−9^ mutations per generation [2].

Usually, while assessing the practicality of a Y-STR amplification technique in forensic science, two main factors are considered: mutation rates and discriminatory capacity (DC). Y-STRs can be categorized based on their mutation rates into three groups: slow mutations (SM, <1.0 × (10^−4^)), moderate mutations (MM, 1.0 × (10^−4^) ∼ 1.0 × (10^−2^)), and rapid mutations (RM, >1.0 × (10^−2^)) [5].

The identification of Y-STRs began in 1992, and since then, scientists have detected over 300 new Y-STR markers [6]. The genetic study of populations has seen a significant transformation in recent years with the extensive use of massive parallel sequencing (MPS) techniques. These approaches enable the genotyping of the sample genome and the discovery of new single nucleotide polymorphism (SNP) markers in different haplogroups of the Y chromosome. These newly discovered SNP markers, known for their high level of informativity, show great potential as tools for studying regional and ethnic genetic groups. Advances in sequencing technologies have significantly improved the amount of detail in population gene analysis. This has enabled researchers to investigate the evolutionary relationships of individual lineages within haplogroups with greater precision [7].

Y-STR genetic markers are utilized in particular scenarios in the field of forensics, such as cases involving sexual aggression in mixed situations (where small amounts of male DNA are found in combination with substantial quantities of female DNA [8]). They are also employed in studying the history of human paternal evolution [9] and in the field of molecular anthropology [10]. Being hemizygotes, Y-STR profiles are primarily distinguished by a solitary allele at each separate locus. Therefore, the presence of numerous alleles at these loci, despite having only one copy, is a clear indication of the participation of many male donors. The Y-STR analysis can help in reconstructing paternal ties but occasionally offers insights into the geographic origin of a male DNA donor, which is valuable in investigations involving missing individuals [11].

Through molecular and cytogenetic biological research, we are able to investigate the existence of a wide range of structural variations in the human Y chromosome, including but not limited to deletions, duplications, and inversions [12].

Considering these factors, in addition to current advancements in sequencing technology, investigations into Y chromosomes continue to be significant in the genomic and post-genomic era. Potential applications cover a wide range of fields, from reconstruction of past male migrations and Y chromosomal variability in ancient DNA to the influence of socio-cultural characteristics on genetic variation and extra-pair paternity behavior among human populations and, of course, in the field of forensics [13].

Bibliometry, as a research method, is used in the field of libraries and information sciences, based on knowledge and the theory of diffusion, and has spread widely to assess scientific progress and paradigm changes. This is achieved by identifying the productivity of publications and analyzing quotations on a specific topic, area, institution, or country [14]. Bibliometric research is an adapted method of systematic review of literature involving the use of quantitative and statistical techniques, such as descriptive statistical analysis, performance analysis, cluster analysis, and scientific mapping. They are applied to bibliographic data, including publications and quotations, to assess and understand the evolution and impact of research in a specific field [15]. By exploring the literature generated by bibliometric instruments, one can obtain a broad and informed perspective on previous research, often providing valuable guidance for future directions of research [14].

Scientific mapping can be used to uncover and emphasize the study framework of a particular discipline, bringing attention to the important themes and topics [15-17]. The analysis and graphic depiction are conducted utilizing a range of methodologies, including the following: (1) knowledge clustering is accomplished in specific domains of interest through citation analysis, which involves identifying publications that have a substantial number of citations; (2) co-citation analysis is used to examine the connections between these cited publications, grouping them into clusters that indicate a shared theme or topic; (3) bibliographic coupling is employed to establish relationships between publications that reference the same bibliographic sources. Clustering articles involves categorizing them based on a shared subject, which is determined by their utilization of identical sources of information; (4) knowledge is classified into clusters based on the frequency of occurrence of keywords together in scientific literature using co-word or co-occurrence analysis; (5) co-authorship analysis examines social relationships in the academic field, such as collaborations among researchers, institutions, and countries; (6) PageRank analysis identifies the primary link between knowledge by examining publications referenced in highly cited works.

The main objective of this study is to provide a comprehensive insight into the literature in the field of population genetics, with emphasis on the analysis of the human Y chromosome through a bibliometric approach and knowledge mapping.

The aims of this study were to identify and display the most often cited publications and references with the highest number of citations, and to highlight recent publications at the national level. By analyzing citations, we discovered publications that had a substantial number of quotations and highlighted the countries that were most productive in this area. A co-citation analysis was conducted to determine the most frequently cited references. Bibliographic coupling has been used to identify the most productive sources of publications, while keyword analysis involved co-occurrence networking for all terms and trend analysis on key topics to emphasize the most commonly used keywords. By analyzing the collaboration of the authors at the country level, the strongest links between different countries were highlighted.

## Review

Materials and methods

Data Collection

The specialty papers were selected from the Web of Science (WOS) database, the Web of Science Core Collection Search category (all editions), in January 2024, following the specifications suggested by the Preferred Reporting Items for Systematic Reviews and Meta-Analyses (PRISMA) protocol [18].

To search for relevant articles on the study of human Y chromosome diversity, the keywords "Y chromosome," "human," "genetic diversity," and "haplotype diversity" were entered into the WOS search engine. The initial search returned a total of 2148 items. Based on the title and summary, all articles were analyzed, selecting only those publications that met the criteria for inclusion in the study.

The inclusion criteria for articles are as follows: (1) publications that have focused on research and exploration of human genetic diversity using Y chromosome analysis; this may include studies investigating genetic variability in different human populations, human evolution, migration of peoples, or other issues relevant to understanding genetic diversity based on the Y chromosome; (2) original articles; (3) publications written entirely in English; and (4) articles published in the period 2014-2023 (included).

The criteria for exclusion are as follows: (1) publications exclusively dedicated to researching genetic diversity in non-human organisms, including animals, plants, fish, or insects; (2) publications exclusively focused on genetic diversity determined by genetic components other than the Y chromosome (mitochondrial DNA (mtDNA), X chromosome, or autosomal); (3) publications focused on areas such as clinical research; (4) studies exclusively analyzing ancient DNA.

After the analysis and selection of publications, relevant information, such as the title, authors, summary, keywords, institutions, references for citation, and other details (full record and cited references), was downloaded for a total of 548 articles.

**Figure 1:**
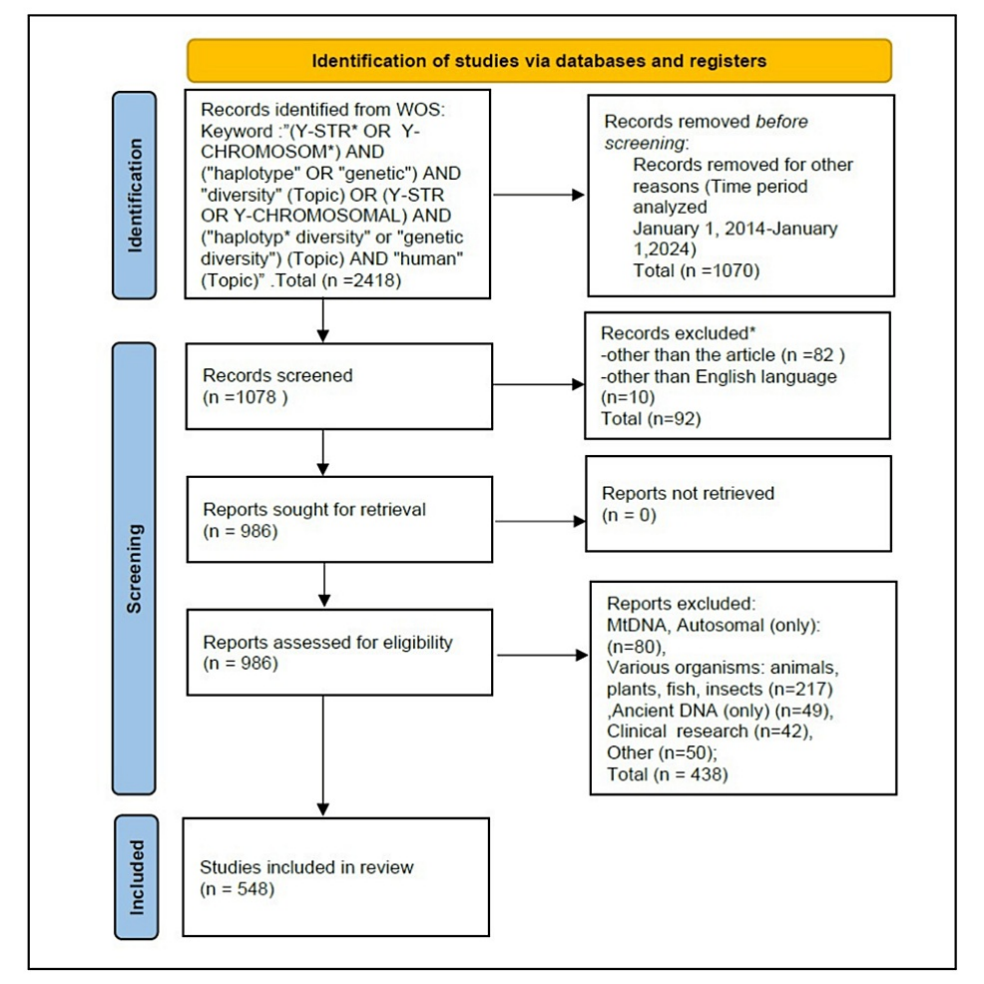
Representation of PRISMA for literature identification in the Web of Science database. PRISMA: Preferred Reporting Items for Systematic Reviews and Meta-Analyses.

Figure 1 presents a synthesis of the approaches used for the identification and analysis of the literature in the WOS database.

Data Analysis

The bibliometric analysis was conducted using the R package "Bibliometrix" and VOSviewer (VOSviewer_1.6.20). The Bibliometrix package is implemented in R (R Foundation for Statistical Computing, Vienna, Austria), an open-source environment that offers a range of tools to streamline quantitative research in bibliometrics and scientometrics [19]. VOSviewer is a cost-free software that enables users to visualize and build bibliometric maps [20].

Results and discussion

General Characterization of Publications and Characterization of Interest Categories

**Figure 2:**
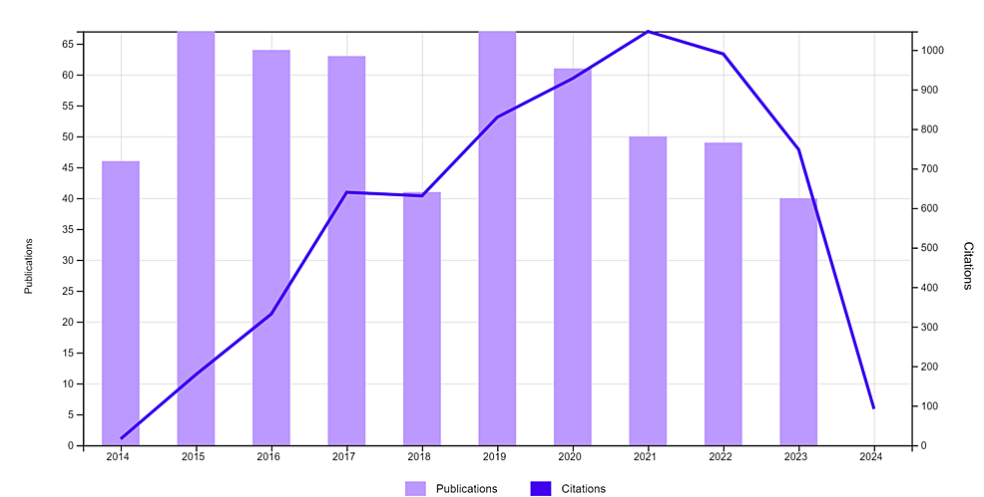
Annual trend of genetic diversity studies based on Y-chromosome analysis 2014–2023 according to the number of citations generated from the Web of Science database.

Of the total of 548 publications analyzed, the most articles (12.23%) were published in 2015 and 2019 (Figure 2). The annual average of publications was 54.8 articles, while the lowest number of articles was recorded in 2018 (7.48%). The annual growth rate for the period 2014-2023 was negative, at -1.01%. A total of 2,982 authors contributed, and the percentage of international co-authors is 47,08%, which shows a special interest in the diversity and genetic heritage of human communities.

Regarding the number of citations included in the study, the average number of citations per document was 11.72, and the articles were cited a total of 6433 times over time (Figure 2). The number of citations without self-citations was 4618. The exclusion of self-citations in the analysis of citations contributes to obtaining a more objective and faithful perspective on scientific production.

According to the WOS categories, the categories that receive the highest level of attention are "Genetics Heredity," "Medicine Legal," and "Anthropology." This indicates a considerable interest in studying genetics, the legal aspects of medicine, and anthropology within the academic and scientific community. These fields contribute to the understanding and investigation of genetic diversity through the analysis of the Y chromosome. There were 309 papers classified in a single category, 182 publications classed in two categories, and 57 publications classified in three categories. Table 1 displays the top 10 WOS categories.

**Table 1:** Top 10 categories in Web of Science (WOS).

No.	Web of Science categories	Record count	% of 548
01	Genetics Heredity	254	46.35%
02	Medicine Legal	172	31.39%
03	Anthropology	80	14.60%
04	Multidisciplinary Sciences	75	13.69%
05	Biology	59	10.77%
06	Biochemistry Molecular Biology	39	7.12%
07	Evolutionary Biology	37	6.75%
08	Public Environmental Occupational Health	35	6.39%
09	Social Sciences Biomedical	16	2.92%
10	Biochemical Research Methods	14	2.55%

Citation Analysis

Citation network for highly cited documents: Through the analysis of quotations, it facilitates the objective identification of influential articles in a specific field and offers the opportunity to explore the connections between the articles that are cited and the ones that make the citations. This analysis highlights the importance of a document based on how frequently it is cited [21]. Therefore, a paper that is frequently cited demonstrates its significance and provides noteworthy and significant contributions to the research discipline.

**Figure 3:**
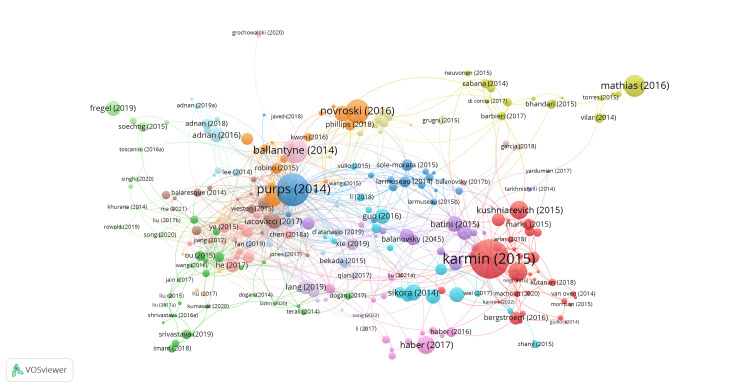
Analysis of citations with VOSviewer for publications with a minimum of five citations and the analysis unit is "Documents."

Figure 3 shows the network of interconnected publications cited at least five times. The citation network shows how by clusters older publications are often used as references in more recent articles, and this contributes to the formation of a network of citations in which the publications cite each other. This network of citations is essential for understanding the connections between the various publications in the field of Y chromosome diversity and for tracking the evolution and interactions between them. The size of the nodes shows the number of citations and the connections between nodes represent the citations.

Upon analyzing the acquired data, it was observed that 67.88% of the documents contained a number of citations between 0 and 10, while 15.51% fell into the range of 10 to 20 citations. Also, 16.61% of the documents were identified to have at least 20 citations. Table 2 details the top 10 articles that have had the most impact in the field of genetic diversity, as measured by the number of citations they have received through the analysis of the human Y chromosome.

**Table 2:** The top 10 most influential articles published in Web of Science in 2014-2023 based on the number of citations on genetic diversity through Y chromosome analysis.

No.	Title	Author(s)	Total citations	Average citations per year	Published year	Reference
01	A recent bottleneck of Y chromosome diversity coincides with a global change in culture	Karmin M et al.	259	25.9	2015	[22]
02	A global analysis of Y-chromosomal haplotype diversity for 23 STR loci	Purps J et al.	196	17.82	2014	[23]
03	Toward male individualization with rapidly mutating Y-chromosomal short tandem repeats	Ballantyne KN et al.	127	11.55	2014	[24]
04	Characterization of genetic sequence variation of 58 STR loci in four major population groups	Novroski NMM et al.	117	13	2016	[25]
05	A continuum of admixture in the Western Hemisphere revealed by the African Diaspora genome	Mathias RA et al.	101	11.22	2016	[26]
06	Human Y chromosome haplogroup N: a non-trivial time-resolved phylogeography that cuts across language families	Ilumäe AM et al.	83	9.22	2016	[27]
07	Genetic heritage of the Balto-Slavic speaking populations: a synthesis of autosomal, mitochondrial and Y-chromosomal data	Kushniarevich A et al.	77	7.77	2016	[28]
08	Continuity and admixture in the last five millennia of Levantine history from ancient Canaanite and present-day Lebanese genome sequences	Haber M et al.	75	9.38	2017	[29]
09	Genetic polymorphisms and mutation rates of 27 Y-chromosomal STRs in a Han population from Guangdong Province, Southern China	Wang Y et al.	72	8	2016	[30]
10	Population genomic analysis of ancient and modern genomes yields new insights into the genetic ancestry of the Tyrolean Iceman and the genetic structure of Europe	Sikora M et al.	65	5.91	2014	[31]

The most cited article in the rankings [22] falls within a wide range of scientific disciplines, such as biotechnology, applied microbiology, genetics, and heredity. It presents the results of a study of 456 sequences of Y chromosomes, which were collected from a wide variety of geographical sources, from unrelated males from 110 different populations. The study confirmed that there was clustering of the major haplogroups of the Y chromosome in a narrow time frame 47000-52000 years ago, which is consistent with a rapid pattern of colonization of Eurasia and Oceania after the narrowing event in Africa.

Most of the rankings explore key issues in the genetic field, including population genetics, medicine, and forensics, focusing on genetic diversity through Y-STR chromosome analysis. One of the most cited articles in the rankings [23] details a global collaboration involving the sampling of 19630 chromosomes from 129 different populations in 51 countries. This study highlighted the discriminatory power of the marker set for 23 Y-STRs, highlighting the same general patterns of population structure as in other marker sets.

On the same subject, the article [24] presents the results of a collaboration between 52 research centers that generated control data for 13 fast-mutation Y-STR loci (RM) in 14644 unrelated males from 111 populations worldwide. This study makes a significant contribution to the field of human genetics, focusing on the use of short Y chromosomal tandem repetitions (Y-STR) with rapid mutation (RM) for the individualization and differentiation of unrelated and related males. Testing a new set of 13 Y-STRs RM on a large scale demonstrates a remarkable ability to individualize unrelated males, with over 99% of them being fully identified. The study also highlights an extremely large haplotype diversity and a rare division of haplotypes between and within populations, which suggests increased effectiveness of this set of markers in individual identification.

In the context of the genetic diversity explored in Y-STR chromosome analyses, a significant study published in 2016 [25] investigated genetic variation in the repeated and flanking regions of a number of autosomal short tandem repeat (STR) markers and X and Y chromosomes. This study, involving 777 unrelated individuals, identified new variation subtypes and analyzed the discriminatory power of STR markers in the context of MPS.

Another study showing genetic diversity [30] examined genetic polymorphisms and mutation rates of 27 Y chromosomal STR locks in a Han population in Guangdong Province, South China. The study, which involved 1,033 father-son pairs and 1,007 unrelated male fathers, aimed to provide data on the haplotypic diversity of the Y chromosome and reliable estimates of mutation rates. Analysis using the Yfiler Plus system (Thermo Fisher Scientific, Waltham, MA) revealed significant haplotype and genetic diversity in the Han population of Guangdong, with observation of 1.003 different haplotypes and identification of varying rates of mutation at different Y-STR locations. These studies bring new insights into the genetic variation of the populations studied and provide essential data for understanding evolution and genetic relationships between different populations.

A relevant study in the category of genetics and heredity [27] examined the paternal haplogroup N in Northern Eurasia with the aim of determining its phylogenetics and phylogeography in a wide range of populations. The study highlighted a rapid expansion of the Siberian sub-clade N3a3'6 in the European direction, with possible historical and archaeological connections.

Another study [29] involved five generations of 3700-year-old Canaanites from Sidon and 99 people from Lebanon to catalog the Levantine genetic diversity. Descent of the Bronze Age Canaanites has been discovered, coming from urban populations on the coast and rural populations, with mixed origins from Neolithic and Eastern Mesopotamian populations. The results showed a significant correlation between the Bronze Age Sidon and Lebanese samples, suggesting a genetic continuity in the region. The study provided information on the genetic history of the Canaanites and their contribution to the current population of the Levant.

A study [31] investigated the results of the genome sequencing analysis of the Tyrolean Iceman mummy, discovered in 1991, as well as more than 400 sardines and two Thracian ancestors from Bulgaria. The aim was to examine the origins and genetic relationships of the Ice Man with modern European populations, using genomic analysis and main component analysis (principal component analysis). The results showed a genetic association between the Ice Man and the Sardinians, suggestions for migration and agricultural expansion in the Neolithic period, and a better understanding of the demographic history of these ancient populations.

Thus, the research presented offers a complex perspective on the evolution and relations between ancient and modern populations, contributing to a deeper understanding of the human genetic past.

A study [26] in the field of multidisciplinary studies aimed to examine the genetic diversity of populations with African origins in North, Central, and South America and the Caribbean. Its objective was to identify genetic variations and contribute to the discovery of genes associated with diseases that affect these populations. The study utilized complete genomic data from 642 individuals representing 15 distinct populations. The findings revealed substantial genetic variation within the African diaspora, characterized by intricate patterns of genetic divergence among different ethnicities. Simultaneously, a separate investigation [28] concentrated on examining the genetic variation among individuals who speak Balto-Slavic languages. This analysis utilized data from more than 6000 DNA and SNP samples encompassing the entirety of the genome. The findings indicated that the Balto-Slavic groups exhibit a high degree of genetic isolation, with clear differences in genetic makeup between the western and eastern regions. These studies highlight the complexity and variety of human genetics, highlighting the importance of multidisciplinary investigations in understanding the historical background and genetic variability among various human communities.

Citation Network for Publication Country

**Figure 4:**
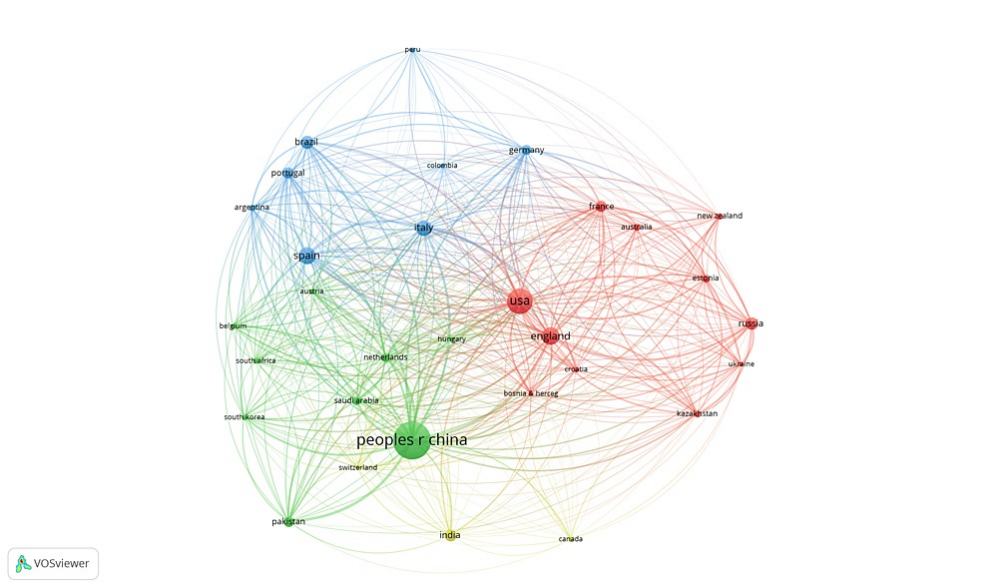
Analysis of citations with VOSviewer for countries that produced at least 10 documents, in the period 2014-2023, on genetic diversity through Y chromosome analysis.

Figure 4 illustrates the analysis of quotations at the country level through a network view. By establishing the network, consideration was given to countries that had produced a minimum of 10 documents. A collection of 31 nations was organized into four groups, each comprising 457 links for a cumulative link strength of 9886. The country with the most citations in the red cluster is the United States, with 30 bonds and a bond strength of 1683. In the green cluster, China has 30 links and a bond strength of 2218. Italy follows with 30 ties and a bond strength of 1211 in the blue cluster. India has the highest number of links in the yellow cluster, at 29 links, with a bond strength of 245.

Table 3 lists the 10 most productive countries in terms of their publications output pertaining to genetic diversity by Y chromosome analysis during the period 2014-2023, according to the WOS. Each country's output is detailed with the number of total citations, average quotations per article, and H-index.

**Table 3:** The list of the 10 most cited countries for the number of publications, in the period 2014-2023, according to the WOS, with the theme of genetic diversity by the analysis of the Y chromosome. Total citations = According to the Web of Science (WOS), this is the total number of citations to all articles in the result set (country). Average per item = According to WOS, this is the average number of articles cited for all articles in the result set (country) and is the sum of the number of citations divided by the number of results in the set. H-index* = According to WOS, the h-index value is based on a list of publications ranked in descending order after the number of times cited, an index of h means that there are h works that have been cited each at least h times.

No.	Countries	Documents	% of 548	Total citations	Average per item	H-index*
01	China	173	31.57%	1,946	11.25	22
02	USA	100	18.25%	2,357	23.57	25
03	England	59	10.77%	1,777	30.12	21
04	Spain	57	10.40%	1,130	19.82	18
05	Italy	51	9.31%	1,163	22.8	19
06	Brazil	39	7.12%	757	19.41	13
07	Russia	38	6.93%	712	18.74	12
08	India	33	6.02%	340	10.3	9
09	France	32	5.84%	886	27.69	15
10	Portugal	31	5.66%	661	21.32	13

The most recent article from China [32] focuses on the development and optimization of a fluorescent labeling kit for forensic purposes; it uses 59 Y-STRs and three Y-indels in the process. The sensitivity, robustness, and discriminatory power of this kit, which has been validated for forensic applications, have been significantly improved in the case of Han Chinese males. This represents an important step forward in the field of molecular genetics and genetic analysis.

A recent genetic study conducted in collaboration with US researchers explored genetic diversity in the Samegrelo (Mingrelia) region of Georgia [33]. Through the analysis of 485 individuals, of which 372 were male participants, the genetic differences between populations in the northern and southern regions were analyzed, and the results from the Y chromosome perspective showed that Mingrelians present a diversity of haplogroups, including E1b1b, G2a, I2, J1, J2, L, Q, R1a, and R1b. The study also revealed genetic similarities between Mingrelians and earlier Georgian genetic populations, as well as the presence of rare Eastern Eurasian haplotypes in the Mingrelian population.

A study published in 2023, in collaboration with researchers in England, analyzed the genetic structure of 379 men in Qatar [34] and showed that the most polymorphic locus was DYS458, with a genetic diversity value of 0.850 and a haplotype diversity of 0.998. The methods used in this study included 23 Y-STR analysis, haplogroup prediction, population genetic structure analysis, and migration rate estimation. The main results included observing the predominance of haplogroup J1 in the population of Qatar, as well as the presence of a distinct genetic structure specific to the populations of the Middle East and Africa. Significant migration routes between Qatar and other countries on the Arab Peninsula have also been identified.

The genealogical and genetic origins of the Spanish regal surname "Castilla" were the subject of the most recent investigation studied in Spain [35]. The study group consisted of participants having the surname "Castilla" along with their corresponding Y chromosome haplotypes. This investigation identified two significant haplogroups, namely, R1b and E1b1b-M81. The analysis revealed the possible presence of an ancestry that includes a significant proportion of the Castilian populace classified under haplogroup R1b. The findings highlighted the complexity and distinctive nature of the Castilla family name's etymology. The association between haplogroup/haplotype and the family name was considered difficult due to historical factors and genetic diversity that were involved.

A study [36] evaluated the effectiveness of increasing the number of markers analyzed and achieving haplotype resolution. For this purpose, the genetic profiles of 115 unrelated individuals from northeastern Italy were analyzed using three different sets that can analyze from 22 to 25 Y-STR and RM Y-STR markers. For each kit, allele frequencies, gene diversity values, and several parameters used in forensics for comparison were calculated. By creating a virtual panel of 30 Y-STRs from the combination of three kits, it was found that the number of external manufacturers does not significantly improve the optimal haplotype resolution.

Another study, conducted in collaboration with Brazilian researchers [37], investigated the genetic relationships between Panoan-speaking and Takanan-speaking communities in South America. This study utilized data obtained from communities located in Llanos de Mojos and other Amazon populations. Comparative analyses of paternal lineage revealed that the Takanan speakers residing in Bolivia and Peru share relatively recent common ancestors; one group had ancestral connections to the Arawakan, Jivaroan, and Cocama, to Panoan speakers, thus confirming the linguistic findings.

A study aimed to analyze the history of the ancient settler population in northern Yakutsk by examining the Y chromosomal lines of three groups of inhabitants in the Russkoe Ust’ye settlement in Yakutia: "Pomors," "Cossacks," and "Zashivertsy" [38]. The findings indicate that the majority (85.7%) of the ancient residents on the Arctic coast of Yakutia, who claim to be descendants of Pomors, have the Y chromosome line N3a4-Z1936 as their predominant genetic marker. This lineage has been discovered mainly in northeastern Europe, specifically in Finland and northern Russia, and it is absent in the nearby indigenous communities of eastern Siberia.

A study [39] was conducted on the Brahmin population of Haryana, India, to generate data on the Y-STR haplotypes. An average gene diversity value of 0.654 was observed for all the loci tested using the analysis of 23 Y-STR markers. The lowest value (0.411) was observed at the DYS391 locus, while the highest values (0.805) were observed at the DYS576 and DYS570 loci. The average haplotype diversity in the Brahmin population is 0.999, while the discriminatory capacity is 0.9696. The dominant haplogroups in this population are R1a, J2a, and L.

Researchers in France have recently published studies that examine genetic diversity in communities in Island Southeast Asia and Oceania by analyzing the Y chromosome. The study [40] examined more than 380 men from Southeast Insular Asia (ISEA) and Oceania. It presented the results of Y chromosome sequencing from complete genomes of 152 samples, as well as phylogenetic analysis and dates. Paternal lineages from ISEA and New Guinea are usually assigned to the haplogroups C-M130, M-P256, S-B254, and O-M175. The results suggest that although there is a lack of data on the chrY sequence in Australia, there is evidence of genetic intersection between certain Australian and New Britain communities, suggesting that there was a certain level of interaction between Australia and the Papuan world.

A collaboration involving researchers from Portugal [41] performed a study on 548 individuals (eight women and 540 men) from eastern Paraguay. These individuals were genotyped for three sets of markers: mtDNA, Y-SNPs, and autosomal AIM-InDels. The purpose of the study was to investigate the impact of various migratory movements on the genetic heritage of the Paraguayan population. By examining the paternal lineages, they discovered a European genetic pool that was very similar to that found in the Iberian populations. There were no substantial differences in the frequencies of haplogroups, suggesting the absence of major events in genetic derivations.

These studies present an accurate and detailed understanding of human genetic diversity, the historical background of populations, and the impact of genetic and historical factors on human diversity and structure.

Co-citation Analysis

**Figure 5:**
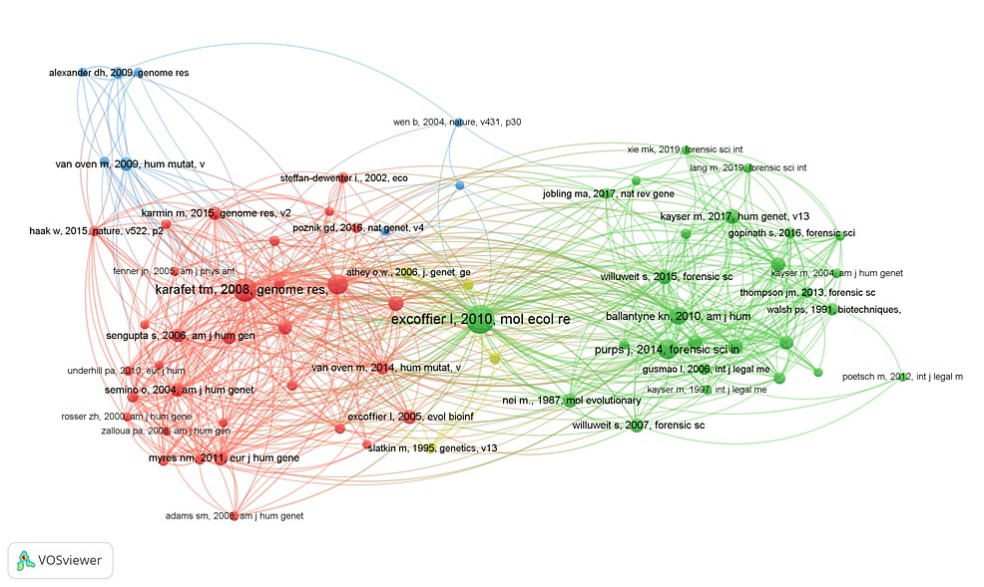
Co-citation analysis with VOSviewer for publications with a minimum of 25 citations.

Co-citation is a scientific technique of mapping that works based on the assumption that publications that are frequently cited together are likely to cover similar subjects. Co-citation analysis is usually used to analyze publication references [42]. Figure 5 illustrates the top 71 publications that are predominantly cited, with a minimum of 25 citations. The distance between the nodes represents the similarity of the subjects discussed, while the size of the nodes corresponds to the number of citations. Each node corresponds to a distinct publication. The node's size corresponds to the number of citations the publication has received; the larger the node, the greater the number of citations. The proximity of the two nodes reflects the degree of similarity between the subjects; the shorter the distance between the two articles, the stronger the correlation.

Four distinct clusters are observed, highlighted by different colors, and the closer two publications are on the co-citation chart, the more similar the subjects dealt with in them, so a smaller distance reflects a closer relationship between them.

Within the green cluster, there are numerous publications that have a significant number of citations. The article with the highest number of citations in this cluster describes a software tool designed for genetic analysis of populations on both Windows and Linux operating systems [43]. Another article from the green cluster has a large number of citations and presents an updated version of the reference database for the Y chromosome haplotype (YHRD 4.0 site) [44].

An article [45] explored various applications of Y chromosome DNA in the field of forensics, including paternal descent identification, testing for paternity, analyzing kinship and family connections, relative male differentiation for individual identification, determining paternal bio-geographical descent, and analyzing potential future applications of Y-chromosomal DNA analysis in criminal investigations.

Two frequently cited articles examined mutation rate estimates of the 13 most mutable Y-STRs in an independent sampling set [46] and explored the potential of rapidly mutating Y-STR markers to differentiate between male relatives and paternal lines, providing significant insights into the field of Y chromosome analysis in forensic genetics and genealogy [47].

A separate study [23] examined a global collaboration to collect and analyze Y chromosomes from various people worldwide, utilizing a defined set of genetic markers. In the green cluster, the most frequently cited articles focused on papers that provided resources or databases for analyzing the Y chromosome in genetics, discussed the application of Y-STR in forensic science, explored RM Y-STR analysis, and conducted the largest study on global genetic diversity. The presence of these reference works emphasizes the significance and applicability of this topic for the scientific and forensic community, highlighting the ongoing requirement for study and development in this domain.

The article that is the most cited in the red cluster provides a detailed presentation of a revised Y chromosome tree, which highlights 311 distinct haplogroups, including two major haplogroups (S and T), and integrates around 600 binary markers [48]. The study describes significant changes in the topology of the parsimony tree, making substantial contributions to understanding the evolution and variety of the Y chromosome throughout human populations. Another article [49] from the same cluster presented the "median joining" method for deducing intraspecific phylogenies, whereas a study [50] focused on analyzing specific characteristics of the human Y chromosome, highlighting its mutations and diversity. Additionally, it investigated the processes of selection and diversity that shaped the evolution of this genetic marker and interpreted the patterns of variety within an anthropological framework. This offers fundamental knowledge on the development and variety of humans, emphasizing the significant contribution of the Y chromosome in the examination of historical events and genetic connections between different populations.

Another relevant article in this cluster presents the first version of Arlequin 3.0 [51] software, which provides essential tools for the analysis of population genetic data. This software package integrates various methods, including the calculation of genetic diversity indices, estimation of allele and haplotype frequencies, tests for bond balance and selective neutrality, as well as a detailed analysis of population structure. It is a valuable resource for researchers studying genetic diversity and human evolution. Most of the articles in the red cluster present details about the phylogenetic structure of the human Y chromosome, including haplogroups and methods used to deduce intraspecific phylogenies, as well as the first version of a software package that integrates several basic and advanced methods for analyzing population genetic data.

The most cited article in the blue cluster presents a comprehensive update of the phylogenicity of the global variation of human mitochondrial DNA, which is based on both the mutations in the coding region and those in the control regions [52]. The study of the diversity of the Y chromosome and mitochondrial DNA provides complementary insights into the evolution and genetic history of humanity.

The yellow cluster's most popular reference article presents a method called R(ST) that assesses population subdivision by analyzing allele frequencies at microsatellite loci [53]. The method has been proposed and validated through the use of computer simulations. Within the framework of a comprehensive sequential mutation model, it has been discovered that R(ST) provides quite imprecise estimations of migration rates and the times of population divergence.

Bibliographic Coupling

Bibliographic linkage refers to the degree of overlap in the reference lists of publications. The greater the number of common references between two publications, the stronger the bibliographic linkage between them [54].

**Figure 6:**
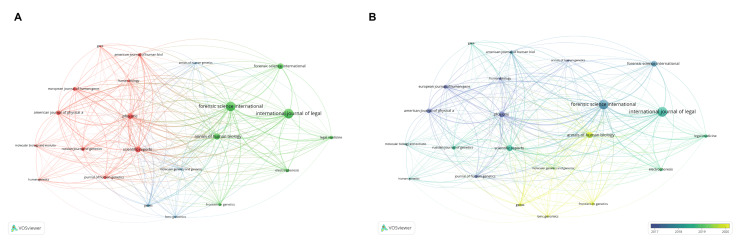
The bibliographic linking network for sources. A. Source-based bibliographic linkage network with a minimum threshold of five documents. B. Source-based bibliographic linkage network with a minimum threshold of five documents represented over time.

Figure 6 illustrates the bibliographic linkage between research sources using the analysis of publications returned by WOS during the period considered. A minimum threshold of five documents has been applied as a criterion for their inclusion in the analysis. This figure highlights the most productive sources by node size, highlighting the close links between frequently cited sources with the same content, viewed by link thickness.

According to Figure 6A, three groups are highlighted: the red cluster, consisting of the American Journal of Human Biology, American Journal of Physical Anthropology, European Journal of Human Genetics, Gene, Human Biology, Human Genetics, Journal of Human Genetics, Molecular Biology and Evolution, PLOS One, Russia Journal of Genetics, and Scientific Reports; the green cluster formed by Annals of Human Biology, Electrophoresis, Forensic Science International-Genetics, Forensic Science International-Supplement Series, Frontiers in Genetics, International Journal of Legal Medicine, and Legal Medicine; blue cluster formed by Annals of Human Genetics, BMC Genomics, Genes, and Molecular Genetics and Genomics.

Figure 6B categorizes the sources based on time period, with yellow tones indicating the most recent sources and dark blue tones representing the older sources. BMC Genomics, a publication of BMC, MDPI's Genes, and FRONTIERS MEDIA SA's Frontiers in Genetics have recently published a series of articles focusing on genetic diversity through the analysis of the Y chromosome.

The main sources for publications on genetic diversity through Y chromosome analysis in the period 2014-2023 are as follows: International Journal of Legal Medicine (13.14%), Forensic Science International-Genetics (12.41%), and Annals Of Human Biology (6.20%). In all three journals, the main themes are as follows: Medicine Legal, Genetics & Heredity, Anthropology, Biology, Public, and Environmental & Occupational Health. The list of the 10 most active journals, with publications on genetic diversity through the analysis of the Y chromosome in the period 2013-2024 and the categories of related topics, is presented in Table 4.

**Table 4:** Top 10 most active journals with publications on genetic diversity through Y chromosome analysis in the period 2014–2023 and related topic categories. * Impact factor, subject category, and category quartile are according to the Web of Science (WOS), Journal Citation Reports 2022.

No.	Journal	Publishers	Number of articles	Impact factor*	Subject category*	Category quartile*
01	International Journal of Legal Medicine	SPRINGER	72	2.1	Medicine, Legal	Q2
02	Forensic Science International Genetics	ELSEVIER IRELAND LTD	68	3.1	Genetics & Heredity	Q2
					Medicine, Legal	Q1
03	Annals of Human Biology	TAYLOR & FRANCIS LTD	34	1.7	Anthropology	Q2
					Biology	Q3
					Public, Environmental & Occupational Health	Q4
04	Scientific Reports	NATURE PORTFOLIO	32	4.6	Multidisciplinary Sciences	Q2
05	PLOS One	PUBLIC LIBRARY SCIENCE	31	3.7	Multidisciplinary Sciences	Q2
06	Forensic Science International Genetics Supplement Series	ELSEVIER IRELAND LTD	30	0.4	Genetics Heredity (WOS Category)	-
07	American Journal of Physical Anthropology	WILEY	22	2.8	Anthropology	Q1
					Evolutionary Biology	Q3
08	European Journal of Human Genetics	SPRINGER NATURE	16	5.2	Biochemistry & Molecular Biology	Q2
					Genetics & Heredity	Q1
09	Russian Journal of Genetics	PLEIADES PUBLISHING INC	16	0.6	Genetics & Heredity	Q4
10	Legal Medicine	ELSEVIER IRELAND LTD	15	1.5	Medicine, Legal	Q3

Co-word Analysis

**Figure 7:**
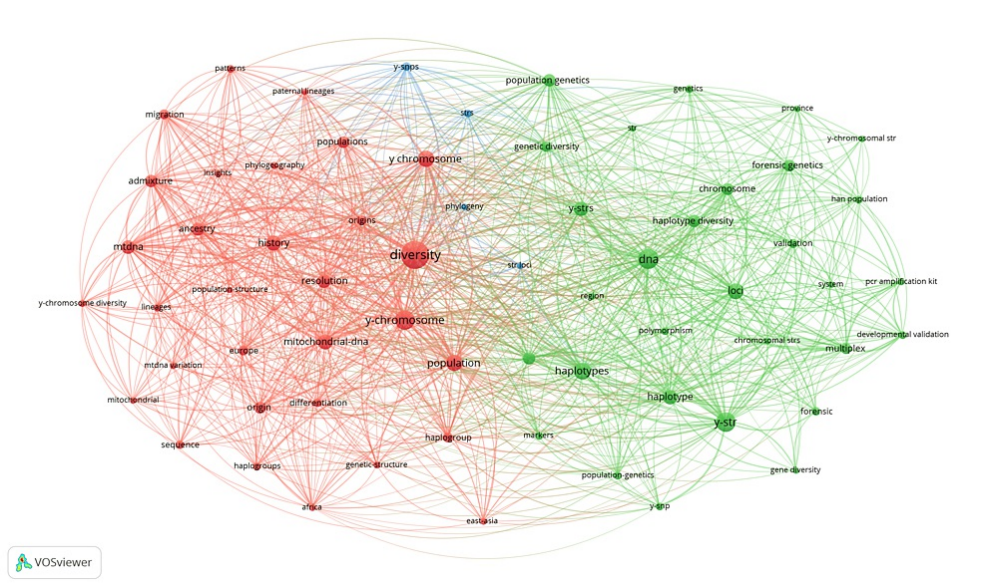
Co-occurrence network of the most commonly used terms together in publications, considering "All keywords" as options.

Co-occurrence network for "All keywords": The co-occurrence analysis applied was "All keywords" as the unit of analysis, which involved both the "Author keyword" and "Keywords Plus." A minimum of 14 appearances was established for the analysis. The map presented in Figure 7 illustrates the clusters into which 65 keywords were identified, making a group of three distinct clusters. Diversity (n = 182), DNA (n = 102), Y-STR (n = 96), Y-chromosomes (n = 96), haplotypes (n = 75), population (n =13), and loci (n = 66) are the words that occur with the greatest frequency.

Analysis of the four clusters shows that all contain the keyword "Short Tandem Repeats" in an abbreviated form or in association with other words. The highest number of references belongs to the green cluster (Y-STR, chromosomal STRs, Y-STRs, STR, STR Loci, STRs, and Y-Chromosome STR), indicating the frequent use of STR analysis in population genetics and forensic genetics for the identification and comparison of genetic profiles. There is also a strong link between DNA and Y-STR within the green cluster. These findings suggest that STR analysis is a common and important method in population and forensic genetics studies, providing an effective way to identify and compare genetic profiles, especially in the context of Y chromosome analyses.

In the red cluster, the most common word is "population," which appears in the forms "Population structure" and "population/s." In this cluster, the most powerful laws are between diversity-Y chromosome (power 41) and diversity-history (potency 36) but there are also strong laws between mitochondrial DNA and diversity (35), suggesting that many studies analyze both Y chromosome and mitochondrial DNA to evaluate genetic diversity or population expansions, highlighting a common concern for understanding the evolution and genetic structure of different population groups.

In the blue cluster, the keywords are grouped as "Phylogeny," "STR Loci," "STRs," and "Y-SNP." It can be noted that this cluster is oriented toward research and studies that focus on phylogenetic analysis, the use of STR sites, and SNPs in the Y chromosome. This association indicates interest in phylogenetic reconstruction and the identification of genetic variation among different populations, for the purposes of analyzing human origin and migration or for investigating genetic relationships between individuals and groups.

Keyword Trend Topics Analysis

**Figure 8:**
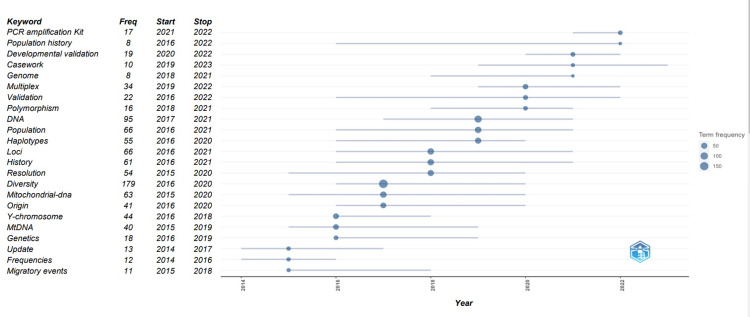
Analysis of the author's keyword trend topics for the period 2014-2023.

Investigating the timing of keywords facilitates a deeper understanding of the directions and topics researched in a field. Figure 8 illustrates the most frequently used group of words for each year in the period 2014-2023, thus providing a detailed insight into thematic developments in population genetics and in the analysis of genetic diversity based on the Y chromosome.

The term "diversity" (n = 179) has the highest frequency in the period 2016-2020, indicating significant attention paid to this aspect in research on the diversity of the Y chromosome. We also note that the terms "mitochondrial DNA" and "origin" occur with significant frequencies in the same range, suggesting links between migration studies and ancestral origins, which may be interconnected with research on the Y chromosome.

In the period 2017-2021, the term "DNA" has the highest frequency, being mentioned 95 times, suggesting significant attention to research involving DNA and its potential in various scientific fields. Also, "population" and "haplotypes" occur with significant frequencies, indicating interest in the study of the structure and diversity of populations as well as the analysis of genetic patterns within a similar timeframe (2016-2021 or 2020).

Another term with a high frequency, i.e., "loci," appears most often (n = 66) in the period 2016-2021. Also, the terms "history" and "resolution" appear with significant frequencies in the same timeframe, suggesting that, in addition to the interest in specific genetic aspects, there is an increased concern for the historical context and improved resolution in the analysis and interpretation of genetic data.

The recent appearance of the term "PCR amplification kit" in the period 2021-2022, along with "population history," suggests that researchers are increasingly interested in modern methods and technologies of PCR amplification while continuing to focus on historical aspects of population evolution, thus reflecting a combination of current and persistent concerns in the field of genetic research.

Co-authorship Analysis (Country Collaboration Network)

**Figure 9:**
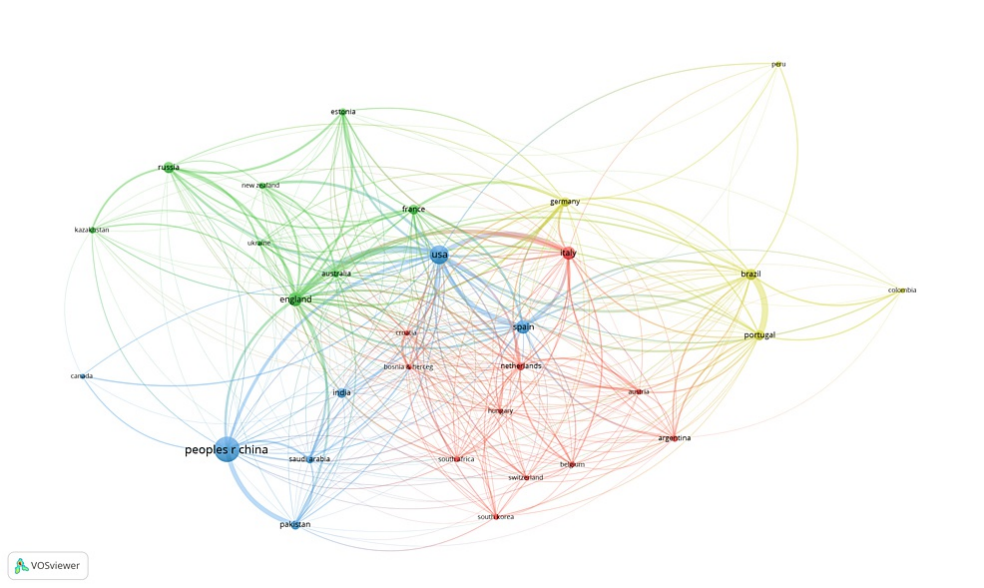
Analysis of collaborations between authors by country with VOSviewer for countries that produced at least 10 documents in the period 2014-2023 on genetic diversity through the analysis of the Y chromosome.

The co-authorship analysis was used to highlight the network of collaborations between countries that produced the most research results. Figure 9 illustrates the network of cooperation between countries in the study of genetic diversity through the analysis of the Y chromosome. The size of the nodes shows the number of publications in a country, and the thickness of the edges that unite the countries shows the strength of collaboration.

For a fixed minimum of 10 publications per country, it is noted that 31 countries are represented in four main groups with a total of 367 links: a predominant group from European countries, including Austria, Belgium, Bosnia and Herzegovina, Croatia, Hungary, Italy, the Netherlands, and Switzerland, but also from other countries in various regions such as Argentina, South Africa, and South Korea (red cluster), a group of majority Asian countries, including China, India, Pakistan, and Saudi Arabia, but also includes countries in other regions such as Canada, the USA, and Spain (green cluster). The third cluster consists of Australia, New Zealand, England, Estonia, France, Kazakhstan, Russia, and Ukraine, and the fourth cluster is represented by countries predominantly from South America: Brazil, Colombia, Peru, Portugal, and Germany.

The distinct geographical distribution of countries in different clusters suggests that there are common collaborations and interests in research between countries in the same cluster. This distribution also indicates a global diversity in research into genetic diversity based on Y chromosome analysis, showing that a variety of countries from different regions of the world are involved in this field and that there is significant global collaboration in research and development.

The strongest link is between the US and England (link straight = 25), but the US also has strong links with Spain and Italy (both, link straight = 17). The latest publication, contributed by researchers from the US and England, presents the results obtained by analyzing the genetic profile of 362 individuals, with at least three generations of local rural descent, chosen to represent the wide geographical spread of the People of the British Isles cohort (PoBI). By analyzing the structure of the biparental population and the uniparental inherited genome segments, it was observed that the Y-STR haplotypes reveal a significant population structure, suggesting Anglo-Saxon migrations to Britain in the 6th century. On the other hand, the motherlines in Britain do not show a significant structure [55].

One of the strongest links is between Portugal and Brazil (link straight = 23). The two most recent publications from Brazil are in collaboration with researchers from Portugal [56] and Spain [57]. Both focus on investigating populations in South America, specifically indigenous males from Ecuador and a population from Tierra del Fuego, Argentina. The study carried out on a batch of 196 unrelated males from Tierra del Fuego Province, Argentina aimed to analyze the diversity of haplotypes and Y haplogroups. Haplogroups within the R clade largely dominate Tierra del Fuego, accounting for almost half of the total data analyzed. The other haplogroups are classified in clusters J, E, I, G, Q, and T, as well as in the KL subgroup. Researchers investigated the probable origin of each of these haplogroups in Eurasia, Native America, and Africa, and found similarities with Italian and Iberian populations in the Tierra del Fuego region, indicating close proximity to the Iberian Peninsula.

Another strong link is between China and Pakistan (link straight = 17). The most recent paper published by researchers from the two countries presents the analysis of genetic diversity in four major ethnic groups in Pakistan [58]. By analyzing 17 Y chromosomal STRs, it was observed that the overall genetic diversity for the Baloch, Pathan, Punjabi, and Sindhi populations ranges between 0.6112 and 0.6657. The results suggest that Pakistani populations do not present a unique set of genes but have genetic affinities with regional populations in Central Asia and northern India.

Collaborations between different countries are significant because they are based on common interests in the field of research. These collaborations strengthen the exchange of knowledge and facilitate progress in the field of population genetics. Historical and cultural links can explain strong collaborations between countries. A common history and mutual cultural influences as well as a common spoken language and neighborliness between countries can facilitate their collaborations in various fields, including scientific research. Thus, history, culture, and geography can play a significant role in establishing and strengthening research collaborations between these countries.

## Conclusions

This article was intended to highlight relevant issues in the field of bibliometrics, based on publications carried out in the period 2014-2023, with the main objective of describing genetic diversity through the analysis of the human Y chromosome. We provide the following information: the most frequently cited publications at both the document and country level, the most frequently cited references, the most commonly used keywords, the sources with the highest number of articles on genetic diversity, collaborations between authors at the country level, and the most significant publications from the past decade. This article can represent a starting point for further research aimed at analyzing genetic diversity using the Y chromosome as a basis. This resource is useful for researchers who are interested in this field, as it provides them with a comprehensive context and an overview of the available literature.
